# Conference presentation to publication: a retrospective study evaluating quality of abstracts and journal articles in medical education research

**DOI:** 10.1186/s12909-017-1048-3

**Published:** 2017-11-09

**Authors:** Christopher R. Stephenson, Brianna E. Vaa, Amy T. Wang, Darrell R. Schroeder, Thomas J. Beckman, Darcy A. Reed, Adam P. Sawatsky

**Affiliations:** 1 Division of General Internal Medicine, Mayo Clinic, 200 First Street SW, Rochester, MN 55905 USA; 2 Division of Biomedical Statistics and Informatics, Mayo Clinic, Rochester, MN USA; 3 Division of Primary Care Internal Medicine, Mayo Clinic, Rochester, MN USA; 4 Harborview, University of California Los Angeles, Los Angeles, CA USA

**Keywords:** Medical education, Medical education–outcomes research, Quality assessment

## Abstract

**Background:**

There is little evidence regarding the comparative quality of abstracts and articles in medical education research. The Medical Education Research Study Quality Instrument (MERSQI), which was developed to evaluate the quality of reporting in medical education, has strong validity evidence for content, internal structure, and relationships to other variables. We used the MERSQI to compare the quality of reporting for conference abstracts, journal abstracts, and published articles.

**Methods:**

This is a retrospective study of all 46 medical education research abstracts submitted to the Society of General Internal Medicine 2009 Annual Meeting that were subsequently published in a peer-reviewed journal. We compared MERSQI scores of the abstracts with scores for their corresponding published journal abstracts and articles. Comparisons were performed using the signed rank test.

**Results:**

Overall MERSQI scores increased significantly for published articles compared with conference abstracts (11.33 vs 9.67; *P* < .001) and journal abstracts (11.33 vs 9.96; *P* < .001). Regarding MERSQI subscales, published articles had higher MERSQI scores than conference abstracts in the domains of sampling (1.59 vs 1.34; *P* = .006), data analysis (3.00 vs 2.43; *P* < .001), and validity of evaluation instrument (1.04 vs 0.28; *P* < .001). Published articles also had higher MERSQI scores than journal abstracts in the domains of data analysis (3.00 vs 2.70; *P* = .004) and validity of evaluation instrument (1.04 vs 0.26; *P* < .001).

**Conclusions:**

To our knowledge, this is the first study to compare the quality of medical education abstracts and journal articles using the MERSQI. Overall, the quality of articles was greater than that of abstracts. However, there were no significant differences between abstracts and articles for the domains of study design and outcomes, which indicates that these MERSQI elements may be applicable to abstracts. Findings also suggest that abstract quality is generally preserved from original presentation to publication.

## Background

Research has shown that only about half of abstracts presented at meetings are subsequently published [1]. Reasons for failure to publish include limited investigator time, insignificant study results, and less rigorous criteria for accepting conference abstracts [2–5]. It is known that the quality of medical education research abstracts and manuscripts is a predictor of journal publication [6]. However, we are unaware of either validated methods for assessing the quality of abstracts or research that compares the quality of medical education conference abstracts with subsequently published journal abstracts and text.

The Medical Education Research Study Quality Instrument (MERSQI) was developed to evaluate the quality of quantitative medical education research studies reported in full-text articles. Previous research established validity evidence for the MERSQI, including content, internal structure (eg, high internal consistency and interrater reliabilities), and relations to other variables evidence such as study funding and correlation with global expert assessments [7, 8]. In addition, the MERSQI has been used to assess the quality of medical education research in systematic reviews [9].

Although the MERSQI has been validated for the assessment of full-text publications, there is little evidence for using the MERSQI to assess the quality of medical education research presented in abstracts. One study demonstrated that abstracts with higher MERSQI scores are more likely to be published [6], but the content and quality differences between abstracts and full journal articles were not evaluated. Abstracts often present incomplete information, possibly because of word-length constraints or pending data at the time of abstract submission [10]. Abstract submission requirements—including word counts and structured versus unstructured formats—also differ between conferences. Increased use of the structured abstract has improved the standardization of content but does not necessarily ensure abstract quality [11, 12].

An instrument to evaluate medical education abstract quality is needed. Therefore, our objective was to compare the overall and domain-specific quality of conference abstracts, journal abstracts, and published articles using the MERSQI.

## Methods

We conducted a retrospective study of medical education research abstracts submitted to the Society of General Internal Medicine (SGIM) 2009 Annual Meeting and subsequently published as abstracts and full-length articles in peer-reviewed journals. For the purpose of this study, we defined *conference abstracts* as those accepted to the SGIM 2009 Annual Meeting and *journal abstracts* as those published along with the final peer-reviewed journal article.

### Study inclusion

**Fig. 1:**
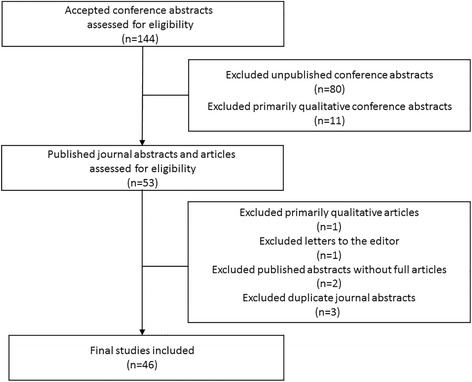
Study Selection. Inclusion and exclusion of medical education abstracts (conference abstracts, journal abstracts, and journal articles) submitted to the 2009 Society of General Internal Medicine Annual Conference

Our dataset was obtained using work from a previous study [6]. A total of 144 medical education abstracts were accepted to the SGIM 2009 Annual Meeting (Fig. 1). Using combinations of author names, keywords, and titles, the authors searched PubMed, ISI Web of Knowledge, and Google Scholar for full-text publications up until December 2013. A total of 64 abstracts were eventually published, with a mean time to publication of 21 months [6]. We included medical education research studies involving educational interventions, curriculum development, assessment tools, and educational surveys at all levels of medical training. Exclusion criteria were based on the exclusion criteria from the original MERSQI validation study [7].We excluded abstracts submitted to the meeting that were never published in a peer-reviewed journal, published letters to the editor, and published abstracts without corresponding full-length journal articles. We excluded abstracts submitted as innovations in medical education or that focused on patient education or biomedical research. In addition, we excluded qualitative research, meta-analyses, and systematic reviews. These article types were excluded in the original MERSQI validation study, and using the MERSQI to evaluate these types of articles would have yielded validity concerns.

Two authors (C.R.S. and B.E.V.) reviewed the 64 conference abstracts, journal abstracts and journal articles for inclusion (Fig. 1). If more than 1 abstract yielded a single publication, we selected the conference abstract that matched the journal abstract’s methods the closest. Disagreements were resolved by author consensus. After review, a total of 46 abstracts submitted to the SGIM 2009 Annual Meeting were included in our study.

### Quality assessment

To assess abstract and article quality, we used the MERSQI, a 10-item tool that evaluates quality in 6 domains: study design, sampling, type of data, data analysis, validity of evaluation instrument, and outcome measures [8]. The MERSQI score ranges from 5 to 18, with higher scores signifying higher quality.

To assess the quality of conference abstracts, 2 authors (A.P.S. and A.T.W.) scored the abstracts using the MERSQI independently and in duplicate. The authors were trained in the use of the MERSQI before the study. All differences were reconciled by consensus, and overall interrater agreement was excellent (interclass correlation coefficient, 0.77–1.00) [2].

Two other authors (C.R.S. and B.E.V.) then used the MERSQI to assess the quality of the corresponding journal abstracts and published articles. These authors also were trained in the use of the MERSQI before the study. Using medical education scientific abstracts not included in this study for calibration, these authors demonstrated high interrater agreement in the use of the MERSQI (interclass correlation coefficient, 0.89), similar to previous studies [9]. Given this substantial agreement, the journal abstracts and articles were divided and reviewed between these 2 authors.

### Statistical analysis

We compared overall and domain-specific MERSQI scores for conference abstracts, journal abstracts, and published articles using the signed rank test. The analysis was performed using SAS version 9.3 (SAS Institute Inc). *P* values less than .05 were considered significant.

## Results

### Overall differences in MERSQI scores between abstracts and articles

Mean total MERSQI scores did not significantly differ between conference abstracts and journal abstracts (9.67 vs 9.96; *P* = .30). However, MERSQI scores were higher for published articles than for conference abstracts (11.33 vs 9.67; *P* < .001) and journal abstracts (11.33 vs 9.96; *P* < .001) (Table 1).

**Table 1:** MERSQI scores and paired comparisons between conference abstracts, journal abstracts, and published articles

	Item^a^			Paired Comparison^b^		
MERSQI Domain (Max Pts)	A	B	C			
	Conf Abs	Jour Abs	Pub Art	B vs A	C vs A	C vs B
Study design (3)	1.54 (0.54)	1.49 (0.55)	1.50 (0.55)	.56	.64	>.99
Sampling	1.34 (0.82)	1.29 (0.70)	1.59 (0.82)	.54	.006	<.001
No. of institutions (1.5)	0.74 (0.43)	0.75 (0.43)	0.75 (0.43)	>.99	>.99	>.99
Response rate (1.5)	0.60 (0.52)	0.54 (0.47)	0.83 (0.63)	.45	.008	<.001
Type of data (3)	2.13 (1.00)	2.39 (0.93)	2.35 (0.95)	.03	.06	>.99
Validity	0.28 (0.62)	0.26 (0.57)	1.04 (1.01)	>.99	<.001	<.001
Internal structure (1)	0.11 (0.31)	0.07 (0.25)	0.26 (0.44)	.63	.09	.01
Content (1)	0.13 (0.34)	0.20 (0.40)	0.59 (0.50)	.45	<.001	<.001
Relationships to variables (1)	0.04 (0.21)	0.00 (0.00)	0.20 (0.40)	.50	.04	.004
Data analysis	2.43 (0.50)	2.70 (0.66)	3.00 (0.00)	.007	<.001	.004
Appropriateness (1)	0.54 (0.50)	0.89 (0.31)	1.00 (0.00)	<.001	<.001	.06
Complexity (2)	1.89 (0.31)	1.80 (0.40)	2.00 (0.00)	.34	.06	.004
Outcomes (3)	1.95 (0.63)	1.83 (0.66)	1.85 (0.67)	.23	.36	.77
Total (18)	9.67 (1.88)	9.96 (2.29)	11.33 (1.82)	.30	<.001	<.001

*Abbreviations:* *Abs* abstract, *Art* article, *Conf* conference, *Jour* journal, *Max Pts* maximum points, *MERSQI* Medical Education Research Study Quality Instrument

^a^Values are mean (SD) score

^b^Signed rank *P* values

### Domain-specific differences in MERSQI scores between abstracts and articles

Domain-specific scores were higher for published articles than for conference abstracts (Table 1). Compared with conference abstracts, published articles had higher MERSQI scores in the domains of sampling (1.59 vs 1.34; *P* = .006), data analysis (3.00 vs 2.43; *P* < .001), and validity of evaluation instrument (1.04 vs 0.28; *P* < .001), specifically the items of content validity (0.59 vs 0.13; *P* < .001) and relationships to other variables (0.20 vs 0.04; *P* = .04) (Table 1).

### MERSQI score differences between journal abstracts and published articles

Compared with journal abstracts, published articles had higher MERSQI scores in the domains of data analysis (3.00 vs 2.70; *P* = .004) and validity of evaluation instrument (1.04 vs 0.26; *P* < .001), specifically the items of internal structure validity (0.26 vs 0.07; *P* = .01), content validity (0.59 vs 0.20; *P* < .001), and relationships to other variables (0.20 vs 0.00; *P* < .004) (Table 1).

### MERSQI score differences between conference abstracts and journal abstracts

Journal abstracts and conference abstracts had subtle differences, although the total MERSQI scores did not significantly differ. When comparing journal abstracts and conference abstracts, journal abstracts had significantly higher MERSQI scores than conference abstracts in the domains of type of data (2.39 vs 2.13; *P* = .03) and data analysis (2.70 vs 2.43; *P* = .007).

### Response rates for abstracts and articles

The MERSQI score for response rate, included in the sampling domain, was significantly higher for published articles than conference abstracts (0.83 vs 0.60; *P* = .008) and journal abstracts (0.83 vs 0.54; *P* < .001). Journal abstracts were less likely than journal articles to report a response rate (8.7% vs 45.7%; *P* < .001).

## Discussion

To our knowledge, this is the first study to use the MERSQI to compare the quality of medical education conference abstracts with their corresponding published abstracts and articles. We found significantly higher quality for published articles than abstracts, with the exception of study design and outcomes, which, independently, may be useful MERSQI domains for assessing abstracts. There were no overall differences in the quality of conference and journal abstracts, which suggests that abstract quality is generally preserved from presentation to publication; however, journal abstracts did score higher for type of data and data analysis. Overall, these findings shed new light on the validity of MERSQI for assessing the quality of medical education research abstracts.

Journal articles had higher overall MERSQI scores than abstracts. This difference was largely related to missing or omitted information in abstracts, including validity evidence, response rates, and appropriateness and complexity of data analysis. We observed low rates of reporting and MERSQI scores for validity evidence among the published articles, which is supported by previous research showing that instrument validity is underemphasized [2, 13, 14]. In addition, response rates and detailed discussions of data analysis were more likely to be reported in articles than abstracts. This may be due to word limits placed on abstracts and/or a lack of awareness among authors regarding the importance of including this information in the abstracts. Greater attention to reporting of response rates and data analysis would improve the quality of abstracts and perhaps the likelihood of abstracts being accepted for presentation.

Reporting of study design and outcomes was not significantly different for conference abstracts, journal abstracts, and journal articles, which indicates that these MERSQI domains may be equally useful for abstracts and articles. Previous studies of medical education research have demonstrated that study design is closely linked to research quality, with randomized control trials being more likely to be published than studies lower on the hierarchy of evidence [1]. In addition, experts have called for study of higher-level outcomes, such as learner behaviors or clinical results [15]. Further research should investigate the usefulness of study design and outcomes as criteria for evaluating abstracts.

Overall quality scores did not differ between the conference abstracts and journal abstracts. However, journal abstracts were more likely than conference abstracts to report type of data (subjective vs objective) and complexity of data analysis (descriptive vs inferential statistics). This may be because data collection or analysis was not completed at the time of presentation or was not as thoroughly discussed at the conference compared with the final presentation. This difference may also be related to more stringent submission requirements for peer-reviewed journals compared with conferences. Our findings reveal that authors could improve conference abstracts, when possible, by providing more detailed explanation of methods, including types of data and how the data were analyzed.

This study provides new validity evidence regarding use of the MERSQI for evaluating the quality of medical education research abstracts. Previous work demonstrated predictive validity regarding the positive correlation between abstract MERSQI scores and subsequent publication [6]. Our study provides fresh validity evidence [16] for using the MERSQI to assess abstracts on the basis of 1) content (based on previous MERSQI content derivation, along with positive support for elements of “study design” and “outcome” and negative support for “type of data” and “data analysis”), 2) internal structure (based on high MERSQI interrater reliability), and 3) relations to other variables based on similarities and differences between MERSQI scores for conference abstracts, journal abstracts, and journal articles.

Our study has several strengths and limitations. To assess study quality, we used the MERSQI, a well-validated tool for evaluating medical education literature. Although there is limited validity evidence for using the MERSQI to assess study quality reported in abstracts, our work helps to guide further use of the MERSQI for abstracts. The authors involved demonstrated substantial interrater reliability that was similar to that in previously published studies [6]. The current study only evaluated medical education research submitted to the SGIM 2009 Annual Meeting. However, because this is a general internal medicine meeting, the abstracts covered a broad range of education content [6]. Although we used a previously researched dataset, it is possible that we missed studies that were published after December 2013 or that studies were missed in the original dataset’s search strategy. In addition, although past research demonstrates that published abstracts have higher MERSQI scores than unpublished, we did not review or compare the MERSQI scores of unpublished articles in our study. Furthermore, the abstract format and length requirements of the SGIM conference may differ from those of other conferences, although we note that MERSQI scores for the conference and journal abstracts did not differ. Last, it is possible that SGIM may attract high-quality research, which could explain the close relationship between MERSQI scores for abstracts and publications. It would be reasonable to study the relationship between abstracts and publications at multiple conferences to ensure that this relationship is not specific to SGIM.

## Conclusions

We found that MERSQI scores of conference and journal abstracts were similar, which indicates that abstract quality is stable from presentation to publication. However, journal articles scored higher than abstracts overall, with the exception of study design and outcomes, which appear to be acceptable domains for rating the quality of abstracts. Differences in quality between journal articles and abstracts existed largely because of missing information in abstracts, including validity evidence, response rate, data type, and data analysis. Attention to these elements, where space permits, would improve abstract quality and may increase the chances of abstract acceptance to meetings. Future research on the utility of a MERSQI modified for abstracts is needed.

## Abbreviations

MERSQI: Medical Education Research Study Quality Instrument
SGIM: Society of General Internal Medicine
